# Brucella bacteremia in patients with acute leukemia: a case series

**DOI:** 10.1186/1752-1947-1-144

**Published:** 2007-11-23

**Authors:** Khalid Ahmed Al-Anazi, Asma Marzouq Al-Jasser

**Affiliations:** 1 Section of Adult Hematology and Hematopoietic, Stem Cell Transplant, King Faisal Cancer Centre, King Faisal Specialist Hospital and Research Centre, PO Box 3345, Riyadh 11211, Saudi Arabia; 2 Section of Microbiology, Department of Pathology, Armed Forces Hospital, Box X-966, PO Box 7897, Riyadh 11159, Saudi Arabia

## Abstract

**Background:**

Brucellosis may cause serious infections in healthy individuals living in countries that are endemic for the infection. However, reports of brucella infections in immunocompromised hosts are relatively rare.

**Case Presentations:**

Reported here are two patients with acute leukemia who developed *Brucella melitensis *bacteremia during their follow up at the Armed Forces Hospital in Riyadh. The first patient developed *B. melitensis *bacteremia during the transformation of his myelodysplasia into acute myeloid leukemia. The second patient developed *B. melitensis *bacteremia while his acute lymphoblastic leukemia was under control. Interestingly, he presented with acute cholecystitis during the brucella sepsis. Both brucella infections were associated with a marked reduction in the hematological parameters in addition to other complications. The bacteremic episodes were successfully treated with netilmicin, doxycycline and ciprofloxacin.

**Conclusion:**

Brucellosis can cause systemic infections, complicated bacteremia and serious morbidity in patients with acute leukemia living in endemic areas. These infections may occur at the presentation of the leukemia or even when the leukemia is in remission. Nevertheless, the early diagnosis of brucellosis and the administration of appropriate antimicrobial therapy for sufficient duration usually improves the outcome in these immunocompromised patients.

## Background

In patients with malignant disorders, infections are major causes of morbidity and mortality. In such patients, the risk of infection is usually related to the intensity and the duration of cytotoxic chemotherapy and immunosuppressive treatment [1]. The main predisposing factors for infections in patients with cancer are: uncontrolled malignancy, immunosuppressive and cytotoxic chemotherapy and immunological deficits that include T-cell depletion and hypogammaglobulinemia. Several immunological defects may be present in these patients, thus making them susceptible to a wide range of opportunistic infections [1].

Brucellosis, the commonest zoonotic infection worldwide, can affect healthy individuals and immunocompromised patients living in countries that are endemic for the infection [2-5]. Two patients with acute leukemia, who developed *B. melitensis *bacteremia during their follow up at the Armed Forces Hospital in Riyadh Saudi Arabia, are reported and the literature is reviewed.

## Case presentations

### Case 1

A 57 year old Iraqi male from Rafha refugee camp was transferred to Riyadh Armed Forces Hospital (RAFH) with pancytopenia for further evaluation and management. He had been experiencing anemic manifestations for two months but no associated bleeding or fever. Physical examination revealed pallor, no external lymphadenopathy or palpable abdominal organomegaly and normal cardiovascular and neurological systems. The complete blood count (CBC) showed: WBC: 1.7 × 10^9^/L, Hb: 35 g/L and PLT: 106 × 10^9^/L. The blood film revealed neutropenia with dysplastic changes and the bone marrow biopsy (BMB) showed a hypercellular marrow with dysplastic changes involving the three hematopoietic cell lines. After establishing the diagnosis of myelodysplastic syndrome (MDS), the patient was given supportive measures to correct his anemia and he was discharged.

Nine months later, the patient was readmitted with high fever, rigors and low backache of one week duration. His physical examination did not reveal any new abnormality. CBC showed: WBC: 1.6 × 10^9^/L with neutrophils of 0.3, Hb: 50 g/L and PLT: 12 × 10^9^/L. The renal and the hepatic profiles were all within normal limits. The blood cultures grew: *B. melitensis *sensitive to ciprofloxacin, netilmicin, and tetracycline but resistant to trimethoprim-sulphamethoxazole (TMP/SMZ). The brucella agglutination antibody titer was highly elevated (1:20480). A repeat BMB showed dysplastic changes and 20% myeloblasts, i.e. evidence of transformation into acute myloid leukemia (AML). For the brucella bacteremia (BB), the patient received IV netilmicin 5 mg/kg three times per day and oral doxycycline 200 mg twice daily for one week. A few days after starting these antibiotics, the fever and the backache subsided. Then netilmicin was replaced by oral ciprofloxacin 500 mg twice daily and the patient was continued on oral doxycycline. After controlling the brucella sepsis, the patient was commenced on an induction course of chemotherapy composed of daunorubicin 50 mg/day IV for 1 day and cytosine arabinoside 100 mg/m^2^/day IV for 5 days. After successful management of both the BB and the leukemic transformation of MDS, the patient was discharged on ciprofloxacin and doxycycline for a total duration of 5 weeks.

Four months later, the patient was readmitted with a new AML transformation of his MDS and severe bronchopneumonia. Cultures of the blood, the sputum and the bronchoalveolar lavage fluid were all negative. There was no clinical or microbiological evidence of recurrence of the brucella infection. However, he received IV netilmicin, ciprofloxacin and amoxicillin but unfortunately he responded poorly to the antimicrobials given and despite receiving full supportive care, he deteriorated further and died.

### Case 2

A 54 year old Saudi male, with history of chronic relapsing brucellosis for 15 years, was diagnosed to have acute lymphoblastic leukemia [ALL] at MD Anderson Cancer Centre in the USA. He achieved the first complete remission (CR) of his leukemia after receiving an induction course of chemotherapy composed of cyclophosphamide, daunorubicin, vincristine, L-asparaginase and intrathecal methotrexate. Three years later, the patient had a central nervous system (CNS) relapse of his leukemia followed by a bone marrow relapse which were treated with intrathecal chemotherapy and three courses of systemic chemotherapy. Subsequently, the patient achieved the third CR of his ALL, but he developed a number of complications including hyperbilirubinemia, generalized pigmentation, pancreatitis, paralytic ileus and hepatic failure. He also developed several infections including staphylococcal bacteremia, *Hemophilus influenzae *bronchopneumonia and *Campylobacter *bacteremia which were all managed successfully.

Five years after the diagnosis of ALL, the patient was readmitted to RAFH with fever for 5 days and abdominal pain of one week duration. Physical examination revealed an unwell middle aged male, generalized pigmentation, a tinge of jaundice, no palpable lymph nodes, a clear chest and normal cardiovascular and neurological systems. The abdomen was distended with tenderness in the epigastrium and the right hypochodrium. The liver and spleen were impalpable, the bowel sounds were positive and there was no ascites. CBC showed: WBC: 2.1 × 10^9^/L (neutrophils: 1.04), Hb: 113 g/L and PLT: 22 × 10^9^/L and the blood film showed no blast cells. The BMB showed a hypocellular marrow with no evidence of leukemia. An abdominal ultrasound showed an acutely inflamed and distended gall bladder. Blood cultures grew *B. melitensis*, sensitive to ciprofloxacin, netilmicin and doxycycline. The brucella agglutination antibody titer was elevated [total 1:2560, IgG 1:640, IgM 1:10240]. The acute cholecystitis was managed conservatively with bowel rest, IV fluids and analgesics. The BB was treated with IV netilmicin 15 mg/kg/day in three divided doses and oral doxycycline 200 mg twice daily for 3 weeks and then the IV netilmicin was replaced by oral ciprofloxacin 500 mg twice daily. After controlling the acute cholecystitis and the BB, the patient was discharged on oral ciprofloxacin and doxycycline for three more weeks. Thereafter the patient had regular follow up at the hematology outpatient clinic and no further recurrence of his brucellosis has been encountered.

## Discussion

Brucella species are small Gram-negative, aerobic and non-motile intracellular coccobacilli that can be isolated from the genitourinary tracts of many wild and domestic animals [2]. The human pathogens: *Brucella abortus *(*B. abortus*), *B. suis*, *B. canis *and *B. melitensis *can cause systemic infections which may affect any body organ [2,6-8]. Over the past decade, the epidemiology of human brucellosis has drastically changed due to a number of sanitary, socioeconomic and political reasons [3]. Several traditionally endemic areas including France and several countries in Latin American have achieved control of the disease while new foci have emerged in Asia and in the Near East [3]. Unfortunately, the disease is still present in varying trends in some European countries and in the USA [3]. Brucellosis is transmitted to humans by direct contact with infected animals or ingestion of unpasteurized milk and dairy products [2,9]. The occupational exposure of abattoir workers, veterinarians and laboratory technicians can result in transmission of the disease through contaminated aerosols [2]. Transmission of the infection by blood transfusion is possible but very unusual [9].

The clinical manifestations of brucellosis and BB are variable and may include fever, rigors, anorexia, malaise, weight loss, backache, bony pains, arthritis and arthralgias and hepatosplenomegaly [2,4-8,10-13]. Brucellosis has been reported to cause acute abdominal conditions such as cholecystitis, appendicitis and pancreatitis [14]. In such conditions, abdominal ultrasound is helpful in establishing the diagnosis and the management includes not only antimicrobial therapy for brucellosis but also specific therapy for the acute abdomen that may involve surgical intervention [14].

Brucellosis may cause a wide range of hematological abnormalities including anemia, leucopenia, thrombocytopenia, pancytopenia, bleeding diathesis and disseminated intravascular coagulation (DIC) [6,8,10-12]. Up to 87.5% of patients with pancytopenia induced by brucella infection have positive blood cultures for the organism and almost 100% of these patients have positive brucella serology [2,6,7]. Almost all the changes in the hematological parameters encountered in patients with brucellosis are transient and reversible provided an appropriate antimicrobial therapy is rapidly instituted [6,11,12]. The bone marrow changes seen in patients with brucellosis are variable and may include: hypercellular or nomocellular marrows, hemophagocytosis and granulomatous changes [6,10-13].

BB develops in 38 to 90% of patients infected with brucella and most of the patients with BB have positive brucella serology [2,7]. BB may be complicated by: infective endocarditis, fatal endotoxic shock that may be associated with DIC, multiorgan failure, microangiopathic hemolytic anemia with bleeding tendency and pancytopenia and death [7,8,13]. Despite the severity of these complications, the early use of appropriate antimicrobial therapy usually leads not only to clinical improvement but also to normalization of the hematological parameters and the coagulation profile [7,8,13].

Although a presumptive diagnosis of brucellosis can be made by demonstrating high or rising antibody titers to brucella antigen, the isolation of the organism from blood, bone marrow or tissue cultures provides the only definitive evidence of the infection [2]. Because of the suboptimal recovery rate of brucellae from the blood, cultures of the bone marrow, liver or lymph nodes may improve the recovery rate of the organism [2]. The use of aerobic bottles of the automated continuous-monitoring blood culture system eg BACTEC 9000 makes possible the diagnosis in more than 95% of positive blood cultures within 7 days [2]. The brucella ELISA test is a rapid test that can be performed easily and it can also be automated. It is a reliable and a sensitive test in the diagnosis of brucellosis [15].

Different antibiotic regimens have been employed in the treatment of brucellosis including the following in various combinations: TMP/SMZ, rifampicin, doxycycline, ciprofloxacin, gentamicin and streptomycin [4,7,8,13]. The mean duration of treatment is usually 6 weeks, but in case of complications like infective endocarditis or spinal involvement, therapy may be prolonged for up to 3 months [7]. The in-vitro resistance to TMP/SMZ is rather high, approximately 29%, but the overall rate of clinical relapse is rather low, about 5% [7].

Brucellosis is one of the leading infections causing pyrexia of unknown origin (PUO) in some parts of the world [4]. In patients with febrile neutropenia who are unresponsive to empirical antibiotic therapy, brucellosis may lead to treatment failure due to the late appearance of signs and symptoms and because of its slow growth in blood cultures [4]. On a few occasions, brucellosis has been encountered in patients with acute leukemia and solid tumors [4,5]. As reported cases of brucellosis developed in patients with malignant disorders living in countries that are endemic for this infection, the dominant presenting features of brucellosis were febrile neutropenia and pancytopenia [4,5]. The first patient presented in this case report developed BB when his primary disease (MDS) was transforming into AML. His blood counts were decreasing further and he presented with severe pancytopenia and the classical features of brucellosis. However, he responded very well to the treatment given, his clinical status improved and his blood indices increased significantly after the control of the brucella sepsis, which allowed the administration of cytotoxic chemotherapy to control his acute leukemia. The second patient had a reactivation of an old brucella infection following the suppression of immunity after receiving repeated courses of cytotoxic chemotherapy to control relapsing ALL. However, this patient developed BB, which was associated with severe pancytopenia, whilst his ALL was under control and simultaneously he presented with clinical and ultrasonic evidence of acute cholecystitis causing an acute abdomen. Both the BB and the acute abdomen were managed successfully despite his age, his poor general condition and the poor prognosis of his primary hematological disorder. Interestingly, both patients had strongly positive brucella serology.

In comparison with the few reported cases of acute leukemia and brucellosis, our patients demonstrated the following peculiar features: (1) The development of BB not only at the presentation of leukemia but also when leukemia was in remission. (2) The profound reduction in blood indices caused by the BB was reversible once an appropriate therapy had been administered. (3) Despite their depressed immunity, both patients were able to mount rather brisk immunologic responses to the BB reflected by the greatly elevated agglutination antibody titers.

## Conclusion

Brucella infection should always be included in the differential diagnosis of febrile neutropenia, pancytopenia and even acute abdomen in immunocompromised hosts living in geographic areas that are endemic for brucellosis. Specific investigations including blood cultures for brucella and brucella serology should be taken and appropriate antimicrobial therapy should be initiated promptly.

## Competing interests

The author(s) declare that they have no competing interests.

## Authors' contributions

Both authors participated, from the clinical and laboratory point of view, in the management of the patients presented as inpatients during their hospitalizations and at the outpatient clinic during their follow up. Both authors read and approved the final form of the manuscript.
